# Supplementary material for: Time-Course Changes in Oxidative Stress and Inflammation in the Retinas of rds Mice: A Retinitis Pigmentosa Model
Source: Antioxidants (Basel). 2022 Sep 29;11(10):1950. doi: 10.3390/antiox11101950 (PMC9598580; doi:10.3390/antiox11101950)

Supplementary table 1. Statistical differences between the different control groups and ages for all the studied parameters.

Area		Number of cell rows in the ONL	TUNEL	Rods	Cones	Avidine	nNOS	GFAP
		Vs. Other Control Ages	Vs. Other Control Ages	Vs. Other Control Ages	Vs. Other Control Ages	Vs. Other Control Ages	Vs. Other Control Ages	Vs. Other Control Ages
Far periphery	11	#	&&					++
	17	#						¡
	21							¡
	28							##
	35							$
	42							
Mid periphery	11		%					¡
	17							%
	21							¡
	28							###
	35							$
	42							
Nerve	11							
	17							
	21							
	28							
	35							
	42							
Total	11	$	$$	&&				%%
	17	$		&			##	¡
	21		$					¡
	28		$					###
	35		$					$
	42							

Area	Age (Day)	Number of branches / Number of cells (Iba1)	Length of branches / Number of cells (Iba1)	CD68
		Vs. Other Control Ages	Vs. Other Control Ages	Vs. Other Control Ages
Far periphery	11			
	17			
	21			
	28			
	35			
	42			
Mid periphery	11			
	17			
	21			
	28			
	35			
	42			
Nerve	11			
	17			
	21			
	28			
	35			
	42			
Total	11	&&	€€€	
	17	€	€	
	21	¡	¡	
	28	$	$	
	35	$		
	42			

Area	Age (Days)	4-HNE	GSH-GSSG
		Vs. Other Control Ages	Vs. Other Control Ages
GCL	11		€
	17		€
	21		€
	28		###
	35		
	42		
IPL	11		
	17		
	21		
	28		
	35		
	42		
INL	11		€
	17		€
	21		€
	28		###
	35		
	42		
OPL	11		+
	17		€
	21		€
	28		###
	35		
	42		
ONL	11		
	17		
	21		
	28		
	35		
	42		
SL	11		
	17		
	21		
	28		
	35		
	42		

Iba1	Age (Days)	Far periphery		Mid periphery		Nerve		Total	
		Vs. rds	Vs. Other Control Ages	Vs. rds	Vs. Other Control Ages	Vs. rds	Vs. Other Control Ages	Vs. rds	Vs. Other Control Ages
GCL	11	*	&&&	*	&&&	*	&&&	*	&&&
	17	*	&&&		&&&				&&&
	21	*	##	*	##	*		*	##
	28	*		*		*		*	
	35	*		*		*		*	
	42	*		*		*		*	
IPL	11		%%%		%%%	*	%%%		@
	17	*	$	*		*		*	
	21	*	$	*	$	*	$	*	$
	28	*	$	*	$	*	$	*	$
	35	*				*		*	
	42	*						*	
INL	11		@@	*				*	
	17	*		*	&	*		*	
	21	*		*		*		*	
	28								
	35								
	42								
OPL	11		%%	*	%%	*		*	%%
	17		%%		%%				%%
	21	*	%%	*	%%	*		*	%%
	28	*	%%	*	%%	*		*	%%
	35	*	$	*		*		*	$
	42	*						*	
ONL	11								
	17	*						*	
	21	*		*		*		*	
	28			*		*		*	
	35			*		*		*	
	42			*		*		*	
Total	11	*	%%%	*	&&&	*	%%%	*	€€
	17	*		*		*		*	
	21	*		*	$	*		*	$
	28	*		*		*		*	
	35	*		*		*		*	
	42	*		*		*		*	

Sign	Difference vs. other ages					
	**11**	**17**	**21**	**28**	**35**	**42**
**#**			21	28	35	42
**##**				28	35	42
**###**					35	42
**$**						42
**$$**		17	21	28	35	
**$$$**			21			42
**&**			21		35	42
**&&**		17	21	28	35	42
**&&&**			21			
**%**			21	28	35	
**%%**					35	
**%%%**			21	28		
**+**		17		28		
**++**		17	21		35	42
**+++**		17	21			
**¡**		17				
**¡¡**		17	21	28		42
**¡¡¡**		17				
**€**				28		
**€€**				28		42
**€€€**		17		28	35	42
**@**		17	21	28		
**@@**			21	28		42
**@@@**			21		35	

Supplementary Figure S1. Representation of the areas used to quantify the row of nuclei and (immune)fluorescence. Sections I a and b correspond to nerve area; Sections II a and b correspond to mid periphery retina and sections III a and b correspond to far periphery retina. The total measurement of each area is approximately 800 µm.

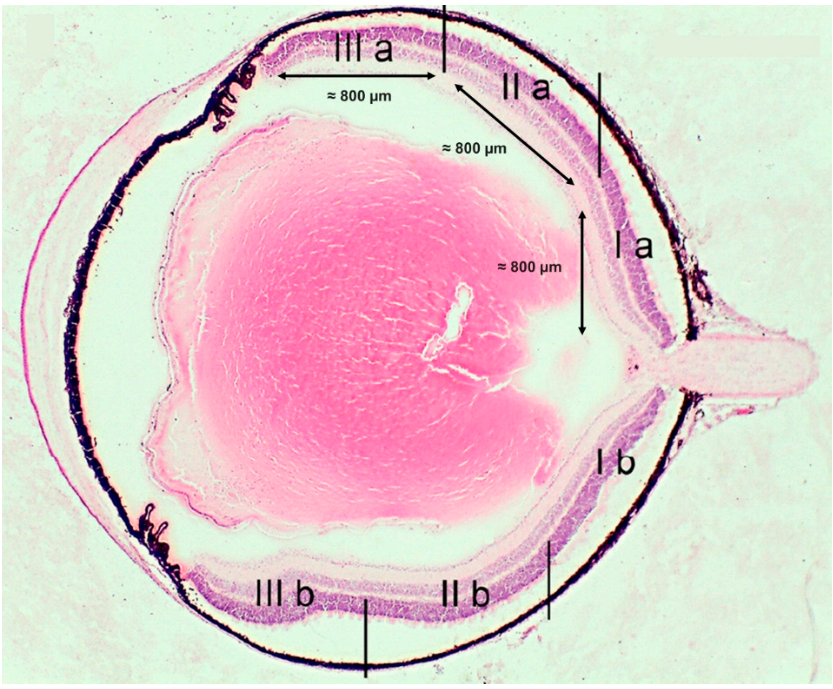

Supplementary Figure S2. Representation of a negative control staining with the secondary antibody Alexa Fluor© 488 goat An-ti-rabbit IgG. Image correspond to a PN17 rds retina.

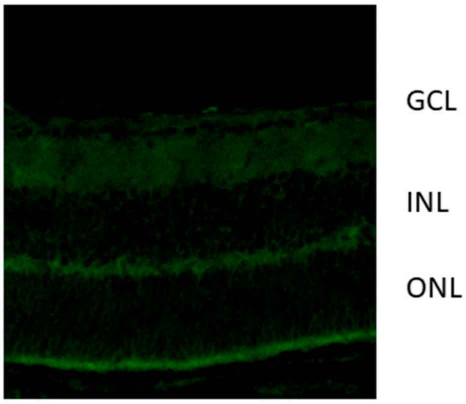

Supplement: Supplementary file 1 [file antioxidants-11-01950-s001.zip › antioxidants-1894656-supplementary.pdf]
